# Supplementary material for: Genomic biosurveillance detects a sexual hybrid in the sudden oak death pathogen
Source: Commun Biol. 2022 May 19;5:477. doi: 10.1038/s42003-022-03394-w (PMC9120034; doi:10.1038/s42003-022-03394-w)
Supplement: Supplementary file 2 — Supplementary Information (new) [file 42003_2022_3394_MOESM2_ESM.pdf]

Genomic biosurveillance detects a sexual hybrid in the sudden oak death pathogen - Supplementary Information

Supplementary table 1. Genotyping assay to assign *Phytophthora ramorum* lineages. Cellulose-binding elicitor of defense in plants and lectin-like (CBEL) genotypes were obtained by qPCR assay to identify *P. ramorum* lineages EU1, EU2, NA1, NA2. The putative hybrid isolates, 16-237-021 and 16-284-032, are homozygous at the CBEL412 locus, a pattern absent in other lineages

	CBEL locus[a]			
Lineage[b]	CBEL245	CBEL349	CBEL373	CBEL412
EU1	G/C	C/T	A/A	C/T
EU2	G/G	C/C	G/G	C/C
NA1	G/G	C/T	A/A	C/C
NA2	G/G	C/T	A/A	C/T
16-237-021	G/C	C/T	A/A	T/T
16-284-032	G/C	C/T	A/A	T/T

[a]The CBEL locus is genotyped at four SNP positions to assign lineages.
[b]For list of additional samples used for genotyping of the three lineages present in North America see Gagnon et al.[1]

Supplementary table 2. *Phytophthora ramorum* genomes used in this study cover locations in Europe and North America and were sampled over more than 20 years.

Lineage	Number of genomes	Geographic origin (number of isolates)	Collection years	Genome coverage[a]
EU1	30	BC, Canada (16), USA (2), Belgium (1), Netherlands (1), France (3), Germany (1), United Kingdom (6)	1995-2017	29.9-78.7
EU2	5	United Kingdom	2007- 2011	38.7-56.5
NA1	29	BC, Canada (5), USA (24)	2001-2017	36.5-100.0
NA2	29	BC, Canada	2004-2017	26.8-92.4
Hybrid	2	BC, Canada	2016	26.8-49.1

[a]Average genome coverage, calculated as N x L/G, where N is the number of reads, L is the average read length and G is the length of the original genome (57.45 Mb).

Supplementary table 3. Observed heterozygosity in *Phytophthora ramorum* lineages and the EU1 x NA2 hybrids

Lineage	Number of genomes	Average coverage (X)[a]	H_o (%)[a,b]	Missingness (%)[a,c]
EU1	30	51 ± 13	35.22 ± 0.05	0.14 ± 0.21
EU2	5	46 ± 6	17.89 ± 0.01	0.22 ± 0.07
NA1	29	70 ± 16	32.48 ± 0.01	0.03 ± 0.03
NA2	29	54 ± 15	31.88 ± 0.03	0.09 ± 0.12
Hybrid	2	38 ± 11	34.59 ± 0.04	3.10 ± 0.94

[a]mean ± standard deviation

[b]Observed heterozygosity: (number of heterozygous sites per individual)/(number of genotyped sites per individual)*100.

[c]Missingness for each genome: (number of missing genotypes per)/(total number of genotyped sites in the vcf)*100.

Supplementary table 4. Genome-wide test of interspecific hybridization among *Phytophthora ramorum* lineages. HyDe inference of hybridization between *Phytophthora ramorum* lineages. P1 and P2 indicate the lineage of the parental lineage and hyb, the hybrids 16-237-021 and 16-284-032. Z-statistic was used to test for significance and *p*-values were adjusted for multiple testing using Bonferroni adjustment. The *γ*-value is expected to be 0.50 in parents to progeny comparisons

P1-hybrid-P2	Z-score (SD)	*p*-value (SD)	*γ*-value (SD)
EU1_hyb_NA2	8.78 (0.24)	0 (0.0)	0.48 (0.02)
NA1_hyb_NA2	3.71 (0.54)	0 (0.0)	0.22 (0.03)
EU1_hyb_NA1	-1.95 (0.18)	0.97 (0.01)	-12.54 (156.51)

Supplementary table 5. Predicted functional impact of SNPs in hybrid *Phytophthora ramorum*. Impact by functional class for variants that generate novel genotypic combinations in the hybrid *P. ramorum* compared to the parents

Variant Impact (functional class)[a]	Counts[b]	Percent
Missense	6,718	35.73%
Nonsense	18	0.10%
Silent	12,066	64.17%
Total	18,802	

[a]Functional impact predicted using SNPeff[2]. Missense are non-synonymous mutations, nonsense are mutations that cause stop codon loss or gain and frameshifts and silent mutations are synonymous (see Cingolani et al.[2] for additional information).

[b]Intralineage polymorphic sites and those with missing data were excluded

Supplementary table 6. Predicted impact type of SNPs in hybrid *Phytophthora ramorum*. Impact by type of variant for SNPs in novel genotypic combinations in the hybrid *P. ramorum* compared to the parents

Variant Impact (Type)[a]	Counts[b]	Percent (%)
High	59	0.03
Moderate	6,693	3.16
Low	12,458	5.89
Modifier	192,346	90.92
Total	211,556	

[a]Type of impact predicted using SNPeff[2]. High impact mutations result in stop codon loss and gain and frameshifts; moderate impact mutations cause a codon to change the amino acid; low modifier mutations are in codon that produce the same amino acid; modifier mutations are in introns, upstream or downstream regions (see Cingolani et al.[2] for additional information and full list of impact types).

[b]Intralineage polymorphic sites and those with missing data were excluded. The totals are larger than the number of SNPs because each SNP can be a modifier of up to five genes upstream or downstream.

Supplementary table 7. Predicted impact of hybridization in gene families associated with pathogenicity. Impact of the mutations was assessed for genes that contained SNPs with novel genotypic combinations in the *Phytophthora ramorum* hybrid (see Table S3). Mutation impact was assessed with SNPeff and reported for members of gene families known to be involved in pathogenicity in *Phytophthora* spp.

Genotypes	Mutation Impact[a]	CAZY[b]	Peptidases	RxLR	CRN
Homozygous for different SNPs in parents, heterozygous in hybrid	high	0	0	1 (1)	0
	moderate	53 (113)	0	19 (41)	4 (5)
Heterozygous in parents, homozygous in hybrid	high	1 (1)	0	0	0
	moderate	25 (55)	11 (21)	23 (52)	4 (5)
Total	-	79	11	43	8
Total predicted in genome	-	332	147	143	31

[a]Impact predicted using SNPeff[2]. High impact mutations result in stop codon loss and gain and frameshifts; moderate impact mutations cause a codon to change the amino acid; low modifier mutations are in codon that produce the same amino acid; modifier mutations are in introns, upstream or downstream regions (see Cingolani et al.[2] for additional information and full list of impact types).

[b]Number of genes (number of SNPs)

Supplementary table 8. New runs of homozygosity (ROH) identified in the *Phytophthora ramorum* hybrid. Regions with ROH enriched (>50%) in homozygous diagnostic[a] SNPs, a pattern unexpected for first generation hybrids.

Scaffold:position	Region size (bp)	No. of homozygous "diagnostic" SNPs[a]	Total No. of SNPs	No. of heterozygous sites[b]	No. of genes[c]	Gene model and putative function[d]		
						5-flanking region	Region of interest	3'-flanking region
Phyram_PR-102_s0001: 1,624,080-1,627,281	3,201	23	25	0 (17; $p < 0.001$)	0 (1; $p < 0.001$)	Folate-biopterin Transporter	-	RxLR effector
Phyram_PR-102_s0001: 3,216,561-3,250,081	33,620	57	58	0 (140; $p < 0.001$)	2 (7; $p < 0.001$)	Flavin oxidoreductase	**DNA binding protein**, Hypothetical protein	Ubiquitin-specific protease
Phyram_PR-102_s0002: 682,333-686,515	4,182	18	18	0 (23; $p < 0.001$)	0 (1; $p < 0.001$)	Hypothetical protein	-	RxLR effector
Phyram_PR-102_s0002: 5,565,957-5,581,249	15,292	37	61	1 (79; $p < 0.001$)	1 (3; $p < 0.001$)	Hypothetical protein	Cyclic nucleotide-gated olfactory channel	Endo-1,3;1,4-beta-D-glucanase
Phyram_PR-102_s0003: 3,689,351-3,694,731	5,380	12	15	0 (28; $p < 0.001$)	0 (1; $p < 0.001$)	Lipase	-	POC1 centriolar A
Phyram_PR-102_s0003: 4,904,972-4,914,539	9,566	43	45	0 (49; $p < 0.001$)	2 (2; ns)	Bidirectional sugar transporter	RxLR effector (x2)	RxLR effector
Phyram_PR-102_s0003: 5,007,985-5,023,658	15,673	29	52	0 (72; $p < 0.001$)	1 (3; $p < 0.001$)	**Mar9 Transposase**	Hypothetical protein	Hypothetical protein
Phyram_PR-102_s0004: 2,194,877-2,213,186	18,309	87	93	0 (82; p < 0.001)	0 (4; $p < 0.001$)	Carbohydrate-binding protein	-	Carbohydrate-binding protein
Phyram_PR-102_s0005: 3,381,122-3,381,801	679	17	21	0 (4; $p < 0.001$)	0 (0; ns)	RxLR effector	-	Hypothetical protein
Phyram_PR-102_s0010: 1,124,549-1,159,669	35,120	80	121	0 (151; $p < 0.001$)	2 (7; $p < 0.001$)	Putative F-box domain-containing protein	Glyceraldehyde-3-phosphate dehydrogenase, **Putative**	Glyceraldehyde 3-phosphate dehydrogenase

							**restriction endonuclease**	
Phyram_PR-102_s0010: 1,778,254-1,813,851	35,597	17	21	0 (152; *p* < 0.001)	0 (7; *p* < 0.001)	RxLR effector	-	Hypothetical protein
Phyram_PR-102_s0013: 769,271-781,265	11,994	76	92	0 (56; *p* < 0.001)	0 (3; *p* < 0.001)	RxLR effector	-	PpiC-type peptidyl-prolyl cis-trans isomerase
Phyram_PR-102_s0014: 331,236-331,563	327	18	18	0 (2; *p* < 0.001)	0 (0; ns)	Hypothetical protein	-	Hypothetical protein
Phyram_PR-102_s0015: 1,370,686-1,401,597	30,911	30	46	2 (159; *p* < 0.001)	1 (6; *p* < 0.001)	Hypothetical protein	Style cell-cycle unhibitor 1	Chaperone protein ClpB
Phyram_PR-102_s0015: 1,401,730-1,446,405	44,675	25	41	0 (229; *p* < 0.001)	2 (8; *p* < 0.001)	Style cell-cycle unhibitor 1	Chaperone protein ClpB,	SH3 domain-containing protein

[a]SNPs that are fixed homozygous for different alleles in the parental lineages NA2 and EU1 and homozygous in the hybrid sample 16_284_032 or SNPs that are fixed homozygous for one allele in one of the parental lineages, homozygous for the alternate allele in the hybrid sample 16_284_032 and fixed heterozygote in the other parental lineage (e.g., 0/0 in NA2, 1/1 in 16_284_032 and 0/1 in EU1).

[b]The expected number of heterozygous sites in a region of same size based on the distribution of heterozygous sites across the genome is in parentheses; the *p*-value is the result of a one sample t-test between the observed value and the distribution of expected values.

[c]The expected number of genes found in a region of the same size based on the distribution of genes across the genome is in parentheses; the *p*-value is the result of a one sample t-test between the observed fixed homozygous for different alleles in the parental lineages NA2 and EU1 and homozygous in the hybrid sample 16_284_032 (e.g., 0/0 in EU1, 1/1 in NA2 and 16_284_032) or SNPs that are fixed homozygous for one allele in one of the parental lineages, homozygous for the alternate allele in the hybrid sample 16_284_032 and fixed heterozygote in the other parental lineage (e.g., 0/0 in NA2, 1/1 in 16_284_032 and 0/1 in EU1). value and the distribution of expected values.

[d]Putative transposable elements are in bold.

Supplementary figure 1. Raw Illumina sequencing read counts at heterozygous loci in the genomes of *P. ramorum* indicates homoploid hybridization*.* The proportion of sequencing reads of hybrid samples 16_237_021 and 16-284-032 for the "minor" allele at heterozygous loci is distributed around 0.5, indicating that a large majority of these loci have only two alleles in equal proportion. Values at the top left of each panel indicate the proportion of loci with read counts consistent with an expected allele ratio of 1:1 (as indicated by a chi-square test of goodness of fit with a critical p-value of 0.05).

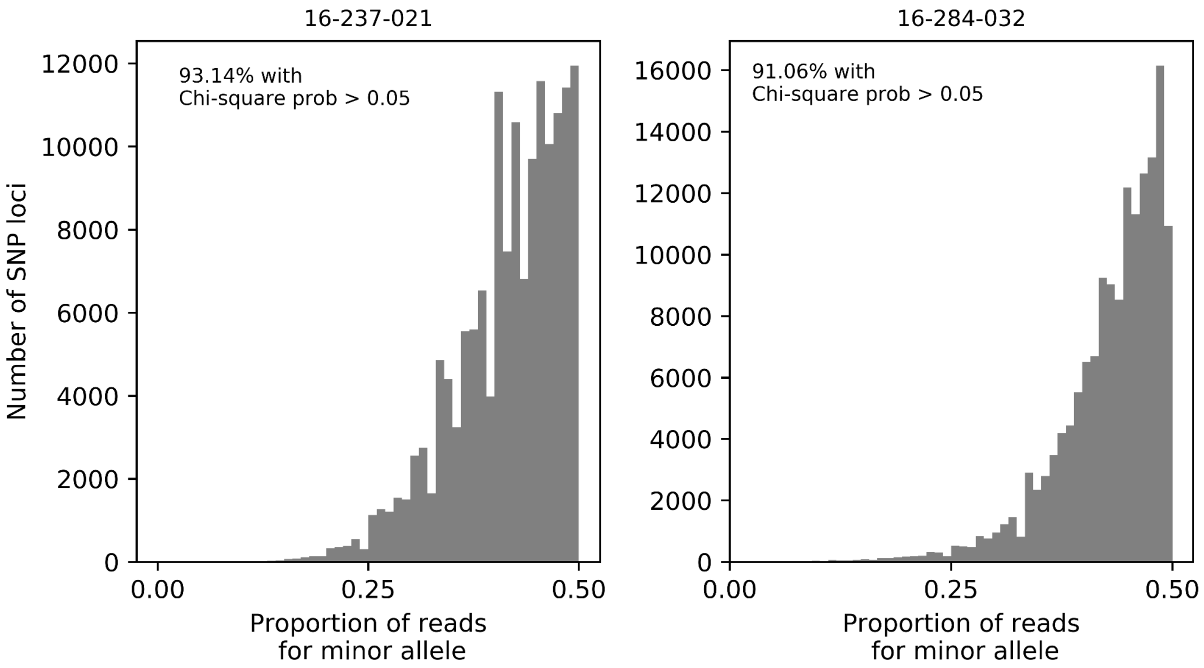

Supplementary figure 2. Assignment of hybrids to *Phytophthora ramorum* lineages. Probability of assignment (obtained from a naïve Bayes classifier) of the hybrid *P. ramorum* sample 16_237_021 to different populations. A, Assignment based on 100 sets of 1,000 randomly selected SNP loci selected along the nuclear genome of *P. ramorum*; error bars (standard deviation) are represented in red. B, assignment based on the 103 SNP loci retrieved from the mitochondrial genome of *P. ramorum*.

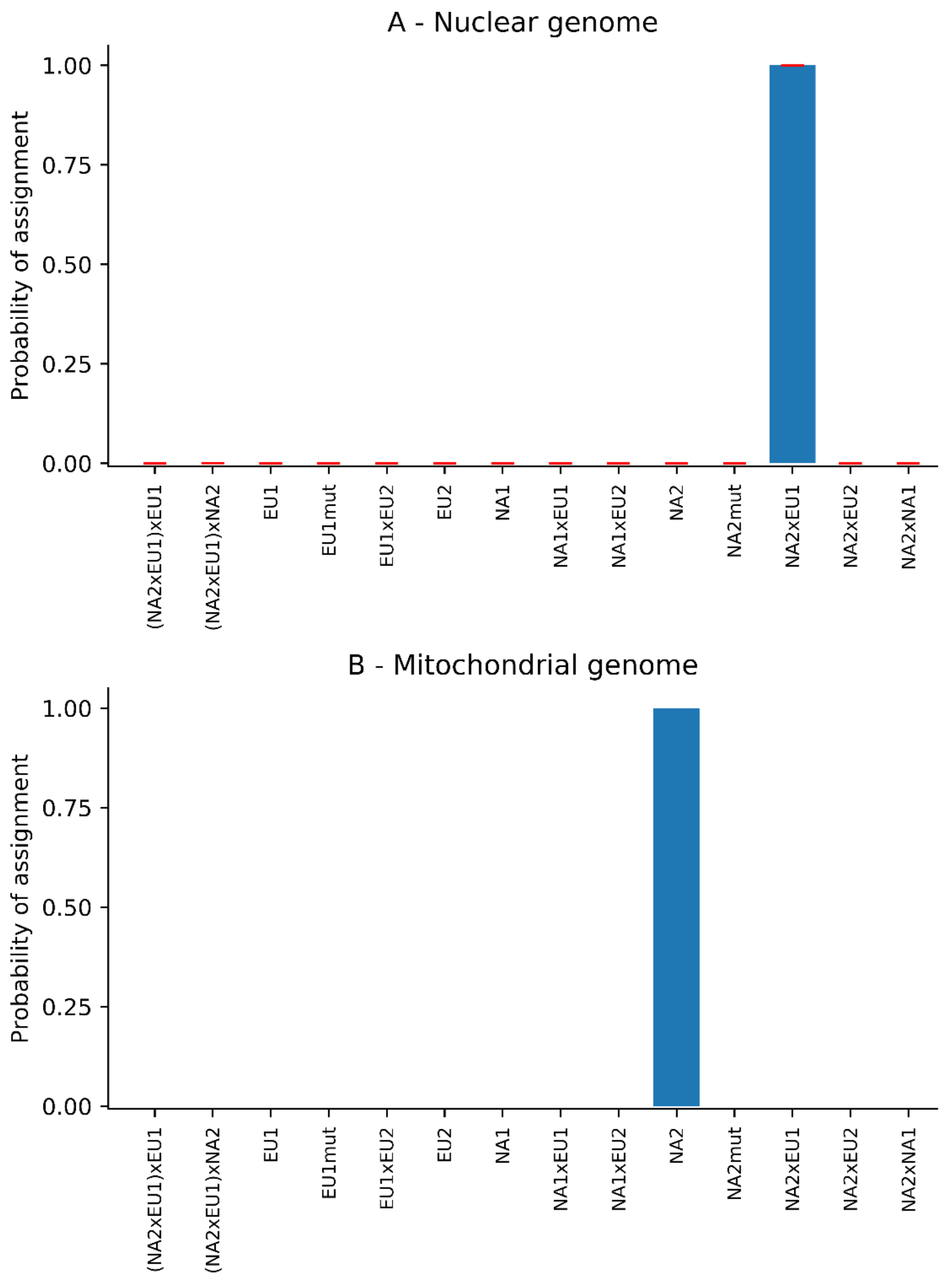

Supplementary figure 3. Phylogenetic tree of hybrid and *Phytophthora ramorum* lineages and simulated populations. Neighbour-joining tree, based on Euclidean distance, of EU1 and NA2 *P. ramorum* isolates, two hybrid samples (Hybrid), 10 simulated F1 hybrids (SimulF1) and 10 simulated backcrosses to each putative parental lineage, NA2 (SimulBCNA2) and EU1 (SimulBCEU1).

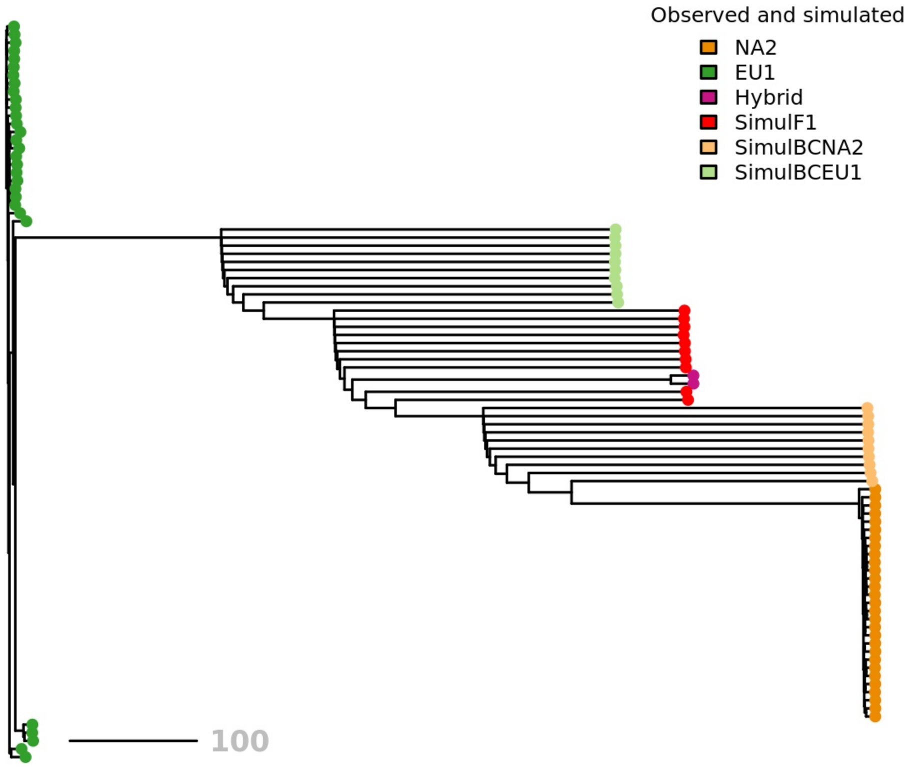

Supplementary figure 4. Mitotic recombination in the *Phytophthora ramorum* hybrid. Distribution of single nucleotide polymorphisms with fixed differences in the EU1 and NA2 lineages that are also homozygous in the hybrid sample 16_284_032 (the other hybrid sample, 16_237_021, had higher proportion of missing values and was not used for this analysis). The 1049 SNPs were sorted into bins of contiguous (1 to >10 SNPs). **A.** Observed distribution of the number of contiguous SNPs in 16_284_032; **B.** Expected distribution of the contiguous SNPs under the assumption of random distribution, with 100 replications. Error bars represent standard deviation

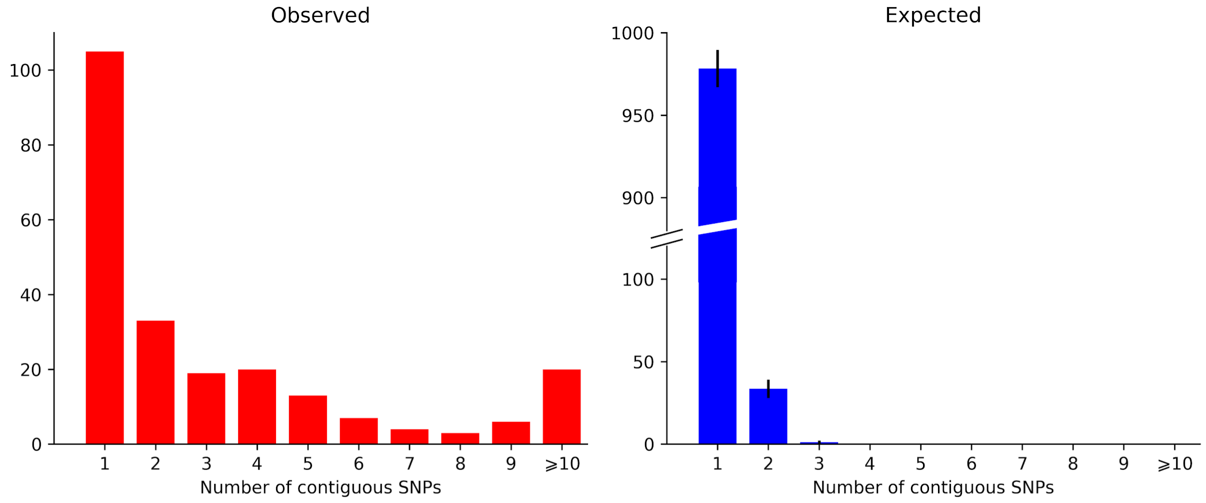

Cited references

1. Gagnon, M.-C., Bergeron, M.-J., Hamelin, R. C., Grünwald, N. J. & Bilodeau, G. J. Real-time PCR assay to distinguish *Phytophthora ramorum* lineages using the cellulose binding elicitor lectin (CBEL) locus. *Canadian Journal of Plant Pathology* **36**, 367–376 (2014).
2. Cingolani, P. *et al.* A program for annotating and predicting the effects of single nucleotide polymorphisms, SnpEff: SNPs in the genome of *Drosophila melanogaster* strain w1118; iso-2; iso-3. *Fly* **6**, 80–92 (2012).
3. Dale, A. L. *et al.* Mitotic recombination and rapid genome evolution in the invasive Forest pathogen *Phytophthora ramorum*. *mBio* **10**, e02452-18 (2019).
4. Yuzon, J., Rizzo, D. M., Tripathy, S., Kasuga, T., & others. Resequencing of the *Phytophthora ramorum* genome to characterize genetic variation and population dynamics of the invasive pathogen. in *Proceedings of the sudden oak death sixth science symposium. Gen. Tech. Rep. GTR-PSW-255. Albany, CA: US Department of Agriculture, Forest Service, Pacific Southwest Research Station: 105-106.* 105–106 (2017).
5. Turner, J. *et al.* Genome sequences of 12 isolates of the EU1 lineage of *Phytophthora ramorum*, a fungus-like pathogen that causes extensive damage and mortality to a wide range of trees and other plants. *Genomics Data* **12**, 17–21 (2017).
