# Supplementary material for: Genomic biosurveillance detects a sexual hybrid in the sudden oak death pathogen
Source: Commun Biol. 2022 May 19;5:477. doi: 10.1038/s42003-022-03394-w (PMC9120034; doi:10.1038/s42003-022-03394-w)
Supplement: Supplementary file 3 — Description of Additional Supplementary Files [file 42003_2022_3394_MOESM3_ESM.pdf]

Description of Additional Supplementary Files

File name: Supplementary Data 1

Description: Metadata associated with the samples collected and sequenced.

File name: Supplementary Data 2

Description: Growth measurements of Phytophthora ramorum hybrids and lineages on carrot agar and lesion size on rhododendron leaves.
