# Supplementary material for: Genomic biosurveillance detects a sexual hybrid in the sudden oak death pathogen
Source: Commun Biol. 2022 May 19;5:477. doi: 10.1038/s42003-022-03394-w (PMC9120034; doi:10.1038/s42003-022-03394-w)
Supplement: Supplementary file 6 — Reporting Summary [file 42003_2022_3394_MOESM6_ESM.pdf]

nature portfolio

Corresponding author(s): Richard Hamelin and Nicolas Feau

Last updated by author(s): Apr 19, 2022

Reporting Summary

Nature Portfolio wishes to improve the reproducibility of the work that we publish. This form provides structure for consistency and transparency in reporting. For further information on Nature Portfolio policies, see our Editorial Policies and the Editorial Policy Checklist.

Statistics

For all statistical analyses, confirm that the following items are present in the figure legend, table legend, main text, or Methods section.

n/a	Confirmed	
☐	☒	The exact sample size (*n*) for each experimental group/condition, given as a discrete number and unit of measurement
☐	☒	A statement on whether measurements were taken from distinct samples or whether the same sample was measured repeatedly
☐	☒	The statistical test(s) used AND whether they are one- or two-sided<br>*Only common tests should be described solely by name; describe more complex techniques in the Methods section.*
☒	☐	A description of all covariates tested
☐	☒	A description of any assumptions or corrections, such as tests of normality and adjustment for multiple comparisons
☐	☒	A full description of the statistical parameters including central tendency (e.g. means) or other basic estimates (e.g. regression coefficient) AND variation (e.g. standard deviation) or associated estimates of uncertainty (e.g. confidence intervals)
☐	☒	For null hypothesis testing, the test statistic (e.g. *F*, *t*, *r*) with confidence intervals, effect sizes, degrees of freedom and *P* value noted<br>*Give P values as exact values whenever suitable.*
☐	☒	For Bayesian analysis, information on the choice of priors and Markov chain Monte Carlo settings
☒	☐	For hierarchical and complex designs, identification of the appropriate level for tests and full reporting of outcomes
☒	☐	Estimates of effect sizes (e.g. Cohen's *d*, Pearson's *r*), indicating how they were calculated

Our web collection on statistics for biologists contains articles on many of the points above.

Software and code

Policy information about availability of computer code

Data collection	*Provide a description of all commercial, open source and custom code used to collect the data in this study, specifying the version used OR state that no software was used.*
Data analysis	The following open source software was used: SAMtools version 1.9, Qualimap version 2.2.1, GATK; version 4.1.0.0, VCFtools version 0.1.16, SNPrelate version 1.28, StAMPP version 1.6.3, Adegenet version 2.1.5, HyDe version 0.4.3, WhatsHap version 1.2, SnpEff Version 4.3. Custom software for the analyses are available at https://github.com/feaunico/SOD_hybrid/tree/v1.0) and Zenodo with https://doi.org/10.5281/zenodo.6465030.

For manuscripts utilizing custom algorithms or software that are central to the research but not yet described in published literature, software must be made available to editors and reviewers. We strongly encourage code deposition in a community repository (e.g. GitHub). See the Nature Portfolio guidelines for submitting code & software for further information.

Data

Policy information about availability of data

All manuscripts must include a data availability statement. This statement should provide the following information, where applicable:

- Accession codes, unique identifiers, or web links for publicly available datasets
- A description of any restrictions on data availability
- For clinical datasets or third party data, please ensure that the statement adheres to our policy

The data that support the findings of this study are included in the paper and all sequence data are deposited in NCBI GenBank as bioprojects, PRJNA791184, PRJNA427329, PRJNA559872, PRJNA177509, and PRJNA558041.

Field-specific reporting

Please select the one below that is the best fit for your research. If you are not sure, read the appropriate sections before making your selection.

☐ Life sciences ☐ Behavioural & social sciences ☒ Ecological, evolutionary & environmental sciences

For a reference copy of the document with all sections, see nature.com/documents/nr-reporting-summary-flat.pdf

Ecological, evolutionary & environmental sciences study design

All studies must disclose on these points even when the disclosure is negative.

Study description	Sequenced the genomes of of 95 isolates of Phytophthora ramorum to determine genomic profile and the level of recombination among lineages.
Research sample	Samples were obtained from the nursery surveys and were collected by co-author, Dr. G. J. Bilodeau. Additional data was obtained from public databases.
Sampling strategy	Samples were collected during inspection of nurseries by collecting leaves with lesions and obtaining pure cultures.
Data collection	Samples were grown on carrot agar and growth was measured. They were also inoculated on rhododendron leaves and the lesion size was measured.
Timing and spatial scale	Samples were collected between 2003 and 2017 in BC nurseries.
Data exclusions	No data was excluded.
Reproducibility	The growth experiments and inoculation experiments were repeated in two different labs and the results were concordant.
Randomization	The assignment procedure was repeated 100 times with random samples.
Blinding	Not relevant

Did the study involve field work? ☒ Yes ☐ No

Field work, collection and transport

Field conditions	Conducted between 2003 and 2017, under different condition. This does not affect the outcome as the goal was to isolate, in pure cultures, the pathogen.
Location	Nurseries in B.C. The metadata is provided in the Supplementary data 1.
Access & import/export	The Canadian regulatory authority, the Canadian Food Inspection Agency, is a collaborator and they have reviewed the manuscript and approved it.
Disturbance	No disturbances.

Reporting for specific materials, systems and methods

We require information from authors about some types of materials, experimental systems and methods used in many studies. Here, indicate whether each material, system or method listed is relevant to your study. If you are not sure if a list item applies to your research, read the appropriate section before selecting a response.

Materials & experimental systems

n/a	Involved in the study	
☒	☐	Antibodies
☒	☐	Eukaryotic cell lines
☒	☐	Palaeontology and archaeology
☒	☐	Animals and other organisms
☒	☐	Human research participants
☒	☐	Clinical data
☒	☐	Dual use research of concern

Methods

n/a	Involved in the study	
☒	☐	ChIP-seq
☒	☐	Flow cytometry
☒	☐	MRI-based neuroimaging
